# Attention/memory complaint is correlated with motor speech disorder in Parkinson’s disease

**DOI:** 10.1186/s12883-019-1535-8

**Published:** 2019-12-01

**Authors:** Ying Liu, Yuchang Gui, Jincui Hu, Shanshan Liang, Sixia Mo, Yuanfang Zhou, Yujian Li, Fengkun Zhou, Jianwen Xu

**Affiliations:** 1 Department of Rehabilitation Medicine, the First Affiliated Hospital of Guangxi Medical University, Nanning, 530021 Guangxi China; 2 Department of Neurology, the First Affiliated Hospital of Guangxi Medical University, Nanning, 530021 Guangxi China

**Keywords:** Motor speech disorder, Parkinson’s disease, Cognitive, NMSS

## Abstract

**Background:**

The mechanisms underlying the online modulation of motor speech in Parkinson’s disease (PD) have not been determined. Moreover, medical and rehabilitation interventions for PD-associated motor speech disorder (MSD) have a poor long-term prognosis.

**Methods:**

To compare risk factors in PD patients with MSD to those without MSD (non-MSD) and determine predictive independent risk factors correlated with the MSD phenotype, we enrolled 314 PD patients, including 250 with and 64 without MSD. We compared demographic, characteristic data, as well as PD-associated evaluations between the MSD group and non-MSD group.

**Results:**

Univariate analysis showed that demographic characteristics, including occupation, educational level, monthly income and speaking background; clinical characteristics, including lesions in the frontal and temporal lobes, and concurrent dysphagia; and PD-associated evaluations, including the activity of daily living (ADL) score, non-motor symptoms scale (NMSS) domain 4 score (perceptual problem), and NMSS domain 5 score (attention/memory) were all significantly different between the MSD and non-MSD group (all *P* < 0.05). Multivariate logistic regression analysis showed that educational level, frontal lesions, and NMSS domain 5 score (attention/memory) were independent risk factors for PD-associated MSD (all *P* < 0.005).

**Conclusions:**

We determined an association between MSD phenotype and cognitive impairment, reflected by low-level education and related clinical profiles. Moreover, attention and memory dysfunction may play key roles in the progression of MSD in PD patients. Further studies are required to detail the mechanism underlying abnormal speech motor modulation in PD patients. Early cognitive intervention may enhance rehabilitation management and motor speech function in patients with PD-associated MSD.

## Introduction

Parkinson’s disease (PD) is a common movement disorder that occurs in more than 1 % of the population worldwide, especially in those over the age of 60 years. PD gives rise to many different kinds of abnormal motor manifestations [1]. As a slow progressing neurodegenerative disease that involves apoptosis of non-dopaminergic neurons, PD imposes various levels of non-motor manifestations (NMM) burden on both young and old individuals, resulting in difficulty in daily activities and social support [2]. Emerging evidence has found that NMM can occur in early or late stage PD and across all stages of life [2, 3]. Moreover, some NMM, including autonomic symptoms, cognitive impairment, mood, and sensory dysfunction, can influence the progression of motor symptoms [4, 5].

Although abnormal motor control of the limbs and trunk is common among PD patients, motor speech disorder (MSD) is also experienced by almost 90% of the patients [6]. MSD, which typically manifests as reduced vocal frequency and volume, as well as abnormal control of the motor aspects of speech, is regarded as a serious impediment to individual psychological health and family care. As a result, MSD is a critical negative determinant of the quality of life of PD patients [7]. Currently, routine speech therapy and medical interventions using anti-PD drugs are the two main therapeutic approaches for PD-associated MSD. In the past, routine speech therapy, including respiratory, voice, and tuning training, as well as rhythmic training, was conventionally carried out for PD-associated MSD. However, the fact that routine speech therapy is mainly aimed at the articulation organs and muscles, rather than the root lesions in the neural substrates responsible for speech motor control, makes it a short-term approach and less effective [8, 9]. On the other hand, given that lesions responsible for PD-associated MSD may not only be found in the basal ganglia, there is mounting evidence that medical interventions are ineffective and may even worsen MSD in PD patients [10–12]. Therefore, the current interventions for PD-associated MSD are still insufficient. This may be attributed to insufficient information on the primary cause of PD-associated MSD, with the pathophysiological mechanisms underlying abnormal motor speech modulation in PD not yet understood.

Speech motor control is predominantly under the online modulation of auditory feedback, which is encoded mainly by cortical areas, especially the frontal and temporal lobes [13, 14]. The frontal and temporal areas are closely associated with high-level cognitive functions, such as attention and working memory, which play important roles in speech motor control [15, 16]. Moreover, several studies have reported that cognitive functions could compensate for auditory degeneration resulting from aging and the diseased brain [17]. Therefore, these high-level cognitive functions may be the target for studies on the pathophysiological mechanism underlying abnormal motor speech modulation as well as the development of novel rehabilitation approaches for PD-associated MSD. Nonetheless, there have been no reports on whether the MSD phenotype is associated with high-level cognitive impairment.

Cognitive impairment has recently been shown to be a risk factor for other kinds of speech disorders (SD) [18], therefore, we speculated that patients with PD-associated MSD might experience a greater NMM burden, especially more severe cognitive impairment. However, there is currently limited evidence on whether the MSD group experiences a greater NMM burden than the non-MSD group or not. To improve the current gaps in the clinical data, we assessed probable independent risk factors, including demographic and clinical characteristics and non-motor symptom scale (NMSS) scores. We aimed to improve the literature on PD-associated MSD and assist future studies on its pathophysiological mechanism. Moreover, we sought to provide information that may help in the development of novel rehabilitation protocols and improvement of current ones.

## Methods

### Patients

We enrolled 314 patients (age range: 42–83 years) with PD. All of them were hospitalized or accepted outpatient service in department of neurology or rehabilitation medicine, within the First Affiliated Hospital of Guangxi Medical University. Two neurologists independently confirmed the diagnosis of PD based on the United Kingdom PD Brain Bank’s definition [19]. The exclusion criteria included: (1) clinically confirmed diagnosis of PD secondary to other diseases; (2) PD concurrent with severe systemic debilitating diseases, such as cardiac, renal, or liver failure; and (3) incomplete case history and neuroimaging data. This study was approved by the Ethics Committee of the First Affiliated Hospital of Guangxi Medical University. We obtained informed consent from each patient.

**Fig. 1:**
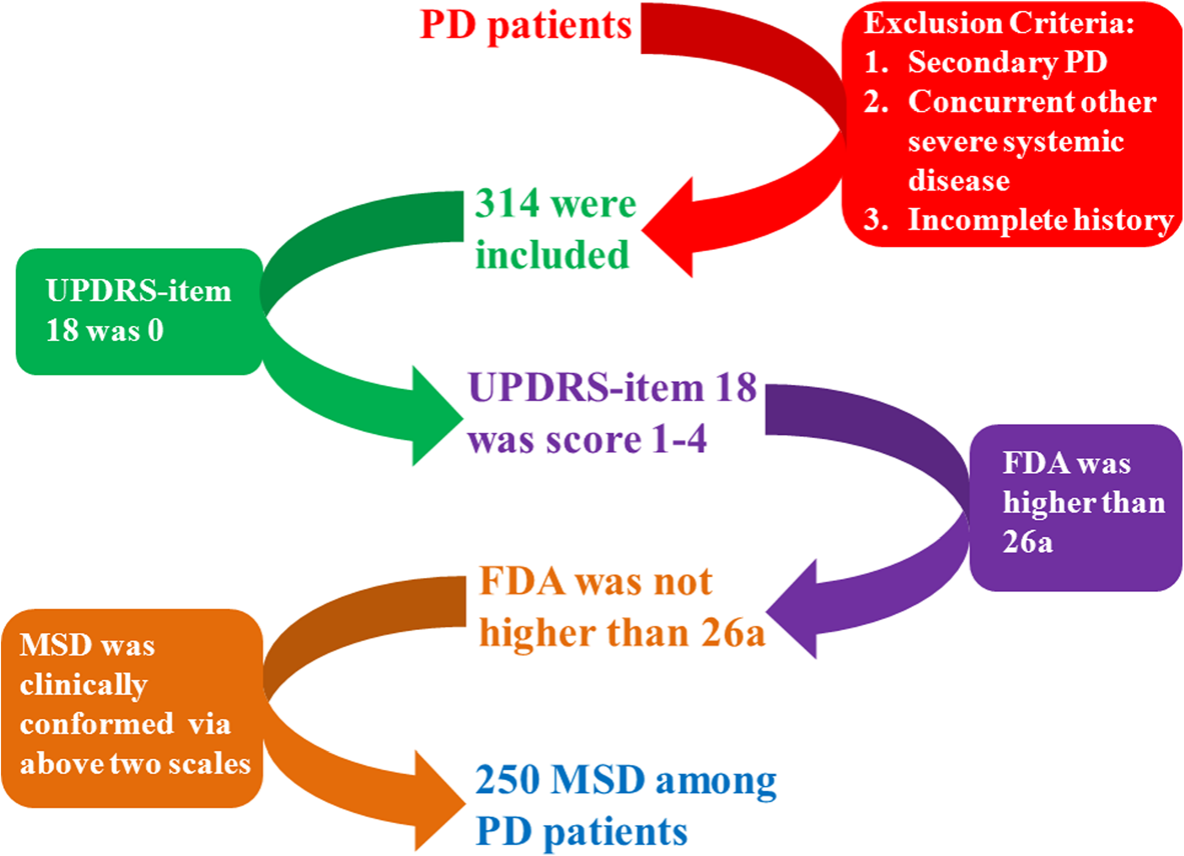
The procedure of patient inclusion

The clinical diagnosis of MSD was confirmed using scores of both the speech (oral communication) item (item 18) of the Unified Parkinson’s Disease Rating Scale (UPDRS) and the Frenchay dysarthria assessment (FDA) by two separate speech therapists. In UPDRS-item 18, score 0 denotes a normal speech motor, Score 1 denotes a mild decline of oral communication and vocal frequency, score 2 denotes to a moderate decline of oral communication and vocal frequency, score 3 denotes to a remarkable decline of oral communication and vocal frequency, and score 4 denotes to a disability of speech motor. Patients with a score 1 or higher were considered as having MSD. In FDA, there are 28 evaluation items. Grade a denotes to a completely normal status. The final evaluated score was the Grade a score and patients with a score of 26a or lower were considered as having MSD. MSD diagnosis was only confirmed when the patient had both an UPDRS-item18 score of one or higher and an FDA score of 26a or lower. Based on these two scales, we assigned the PD patients to either the MSD or non-MSD group. In this study, there were 250 patients in the MSD group and 64 patients in the non-MSD group. The whole diagnostic procedure of MSD is shown in Fig. 1.

### Data collection and scale evaluation

Demographic data were collected via face-to-face interviews or written questionnaires. The mental labor was mainly characterized by application of intelligence, scientific and cultural knowledge and production skills during their labor. And the manual labor was mainly characterized by application of physical strength, production tool and service during their labor.

Clinical data, including case history and magnetic resonance imaging (MRI) brain scans, were collected from the medical records of each patient. Hypertension was defined as the systolic pressure was higher than 140 mmHg or (and) the diastolic pressure was higher than 90 mmHg, or a reported history of hypertension. Diabetes was defined as the fasting blood glucose was higher than 7.0 mmol/L or (and) the 2-h postprandial blood pressure was higher than 11.1 mmol/L, or a reported history of diabetes. Ischemic heart disease was defined as the change of ST-T segment in electrocardiogram plus a manifestation of angina pectoris, or a reported history of coronary heart disease. Lesions in the frontal or temporal lobe were defined as hypointense lesions on T1-weight and/or hyperintense lesions on T2-weight in the frontal or temporal lobe. According to the imaging principle of MRI, only such lesions that exceeded 1 cm in radius were counted. These neuroimaging data was evaluated by our two neurologists independently. Only when the two neurologists concurred that there was lesion in the frontal or temporal lobe could we draw a conclusion.

After obtaining access to each patient’s case history, we evaluated the severity of PD using the modified Hoehn & Yahr staging scale based on the corresponding clinical manifestations. Daily living ability was evaluated via the Modified Barthel Index of activities of daily living (ADL).

We comprehensively evaluated motor function, tremor, and postural instability gait difficulty (PIGD) using the UPDRS motor sum score, tremor score, and PIGD score, respectively [20].

The widely acknowledged NMSS was used to evaluate the degree of the NMM burden [21]. The NMSS contains a total of thirty items that are divided into nine domains that cover different aspects of daily living functions (i.e., cardiovascular disease, sleep/fatigue, mood/apathy, perceptual problems/hallucinations, attention/ memory, gastrointestinal, urinary, sexual function, and miscellaneous). Every item was evaluated quantitatively based on the severity (range: 0 to 3) and frequency (range: 0 to 4) over the previous month. The total score of each item was defined as the domain score. The total score of the NMSS, which is indicative of the overall NMM burden on each patient, was regarded as the sum of the thirty items.

### Statistical analysis

All statistical analyses were carried out using SPSS software (version 18.0). Measurement data and continuous variables are presented as the mean and standard deviation or median and interquartile range, while enumeration data and categorical variables are presented as the frequency and proportion. For univariate analysis, we compared the measurement data from the MSD and non-MSD groups using t-tests or Mann-Whitney *U* tests. Additionally, we used the Chi-squared or Fisher’s exact tests to compare the enumeration data between the MSD and non-MSD groups.

We then carried out multivariate logistic regression analysis to determine the independent risk factors and rule out potential confounding variables. At first, all the factors with *P*-values lower than 0.05 in the t-tests, Mann-Whitney *U* tests and Chi-squared tests were included. To reduce potential family wise error rate (FWER), namely type I error attributable to t-tests and Mann-Whitney *U* tests, a Bonferroni correction of *P*-value was then used for statistical significance in the multivariate logistic regression analysis. After the final logistic regression analysis model, we only considered the risk factors with *P*-values lower than the corrected *P*-value to be the independent risk factors.

## Results

### Demographic characteristics

The demographic data of the two groups are shown in Table 1. There were 250 patients in the MSD group (168 males and 82 females) and 64 patients in the non-MSD group (40 males and 24 females). The average age was 64.3 ± 9.5 years in the MSD group and 62.3 ± 8.7 years in the non-MSD group. Student’s *t*-test analysis showed that there was no significant difference in the average age, age at PD onset, and duration of PD between the two groups (all *P* > 0.05). The proportion of patients with a family history of PD was similar in both groups (*P* > 0.05).

**Table 1:** The comparison of demographic characteristic between MSD and non-MSD groups

	MSD group	non-MSD group	*P* value
Age (years)	64.3 ± 9.5	62.3 ± 8.7	0.088
Age at PD onset (years)	60.5 ± 9.5	58.4 ± 8.6	0.063
Duration of PD (years)	3.9 ± 1.6	4.0 ± 1.6	0.445
PD family history (%)			
Yes	15 (6.00%)	7(10.94%)	0.167
No	235(94.00%)	57(89.06%)	
Gender (%)			
Male	168(67.20%)	40(62.50%)	0.478
Female	82(32.80%)	24(37.50%)	
Marital status (%)			
Married	238(95.20%)	58(90.63%)	0.160
Unmarried	12(4.80%)	6(9.37%)	
Occupation (%)			
Manual labor	170(68.00%)	34(53.13%)	0.026*
Mental labor	80(32.00%)	30(46.87%)	
Educational level (%)			
Higher than bachelor	40(16.00%)	26(40.63%)	<0.001*
Lower than bachelor	210(84.00%)	38(59.37%)	
Speaking background (%)			
Monolingual speakers	199(79.60%)	58(90.62%)	0.041*
Bilingual speakers	51(20.40%)	6(9.38%)	
Monthly income (yuan)			
Higher than 5500	74(29.60%)	28(43.75%)	0.031*
Lower than 5500	176(70.40%)	36(56.25%)	
Leisure activities (%)			
Usually	123(49.20%)	35(54.69%)	0.433
Seldom	127(50.80%)	29(45.31%)	

The “*” mark denotes a significant *P* value (*P* < 0.05) after a Student’s *T* test or a Chi-square test

The Chi-squared test revealed that a higher percentage of patients engaged in manual labor in the MSD group (68.00%) than in the non-MSD group (53.13%; *P* = 0.026). More patients in the MSD group (84.00%) had a low level of education (lack of post-secondary education in university) than patients in the non-MSD group (59.37%; *P* < 0.001). A higher percentage of patients were bilingual speakers in the MSD group (20.40%) than in the non-MSD group (9.38%; *P* = 0.041). More patients in the MSD group (70.40%) had a low income (lower than 5500 yuan) compared to those in the non-MSD group (56.25%; *P* = 0.031). However, there were no significant differences between these two groups in terms of gender, marital status, or leisure activities (*P* > 0.05 for all).

### Clinical characteristics

Data on the clinical characteristics of the two groups are shown in Table 2. There were no significant differences between the two groups in terms of hypertension, hyperlipidemia, ischemic heart disease, or diabetes mellitus (*P* > 0.05 for all).

**Table 2:** The comparison of clinical characteristic between MSD and non-MSD groups

	MSD group	non-MSD group	*P* value
Hypertension (%)			
Yes	172(68.80%)	47(73.44%)	0.471
No	78(31.20%)	17(26.56%)	
Hyperlipidemia (%)			
Yes	94 (37.60%)	21(32.81%)	0.478
No	156(62.40%)	43(67.19%)	
Ischemic heart disease (%)			
Yes	33(13.20%)	5(7.81%)	0.238
No	217(86.80%)	59(92.19%)	
Diabetes mellitus (%)			
Yes	38(15.20%)	12(18.75%)	0.489
No	212(84.80%)	52(81.25%)	
Lesion in frontal lobe (%)			
Yes	195(78.00%)	38(59.38%)	0.002*
No	55(22.00%)	26(40.62%)	
Lesion in temporal lobe (%)			
Yes	129(51.60%)	23(35.94%)	0.025*
No	121(48.40%)	41(64.06%)	
Lesion in parietal lobe (%)			
Yes	2(0.80%)	1(1.56%)	0.576
No	248(99.20%)	63(98.44%)	
Lesion in occipital lobe (%)			
Yes	1(0.40%)	1(1.56%)	0.297
No	249(99.60%)	63(98.44%)	
Concurrent dysphagia (%)			
Yes	116(46.40%)	20(31.25%)	0.029*
No	134(53.60%)	44(68.75%)	
Concurrent other CNS diseases (%)			
Yes	54(21.60%)	18(28.13%)	0.268
No	196(78.40%)	46(71.87%)	
Tremor dominant forms (%)			
Yes	173(69.20%)	41(64.06%)	0.431
No	77(30.80%)	23(35.94%)	
Levodopa medication (%)			
Yes	235(94.00%)	58(90.63%)	0.335
No	15(6.00%)	6(9.37%)	
Dopamine agonist medication (%)			
Yes	237(94.80%)	62(96.88%)	0.487
No	13(5.20%)	2(3.12%)	
Selegiline medication (%)			
Yes	167(66.80%)	39(60.94%)	0.378
No	83(33.20%)	25(39.06%)	
Amantadine medication (%)			
Yes	228(91.20%)	56(87.50%)	0.369
No	22(8.80%)	8(12.50%)	
Levodopa equivalent dose (mg)	396.60	384.40	0.557

The “*” mark denotes a significant *P* value (*P* < 0.05) after a statistical analysis

The Chi-squared test revealed that there were higher incidence of frontal lobe lesions in the MSD group (78.00%) than in the non-MSD group (59.38%; *P* = 0.002). Similarly, there were higher incidence of temporal lobe lesions in the MSD group (51.60%) than in the non-MSD group (35.94%; *P* = 0.025). In addition, a higher percentage of patients had concurrent dysphagia in the MSD group (46.40%) than in the non-MSD group (31.25%; *P* = 0.029). However, no significant differences were found in terms of parietal lobe lesion, occipital lobe lesions, concurrence with other CNS diseases and tremor dominant forms between these two groups (*P* > 0.05 for all).

Anti-PD drugs, such as Levodopa, selegiline, and amantadine, were prescribed more often in the MSD group, while dopamine agonists were prescribed more often in the non-MSD group. However, there were no significant differences in the medication frequency or Levodopa equivalent dose between the two groups (*P* > 0.05 for all).

### PD-associated evaluations

The differences in the PD-associated evaluations between the two groups are shown in Table 3. The Mann-Whitney *U* test revealed that the ADL score was lower in the MSD group than in the non-MSD group (*P* = 0.020). However, there was no significant difference in the Hoehn & Yahr stage score and duration of PD between the MSD group and non-MSD group (*P* = 0.095).

**Table 3:** The comparison of PD-associated evaluations between MSD and non-MSD groups

	MSD group	non-MSD group	*P* value
Hoehn&Yahr stage score (median)	2.0(1.0, 3.0)	2.0(1.0, 2.5)	0.095
ADL score (median)	75(70, 80)	80(70, 85)	0.020*
MDS-UPDRS part III	19.0(10.0, 31.6)	17.0(11.0, 28.8)	0.301
UPDRS tremor score	2.0(1.0, 4.0)	1.5(0, 4.0)	0.263
UPDRS PIGD score	2.0(0.5, 4.0)	1.5(0, 3.0)	0.320
NMSS total score	26.5(14.0, 48.0)	24.5(13.0, 36.0)	0.336
NMSS domain 1 score (cardiovascular)	0(0,1.0)	0(0,1.0)	0.249
NMSS domain 2 score (sleep/fatigue)	3.0(1.0, 8.0)	3.0(1.0, 6.0)	0.606
NMSS domain 3 score (mood/apathy)	4.0(1.0, 12.0)	4.0(1.0, 11.0)	0.743
NMSS domain 4 score (perceptual problem)	3.0(1.0, 5.0)	2.0(0, 3.0)	0.033*
NMSS domain 5 score (attention/memory)	6.0(3.0, 8.0)	1.0(0, 3.0)	< 0.001*
NMSS domain 6 score (gastrointestinal)	1.0(0, 3.0)	1.0(0, 2.0)	0.427
NMSS domain 7 score (urinary)	3.0(0.5, 7.0)	2.0(0, 6.0)	0.126
NMSS domain 8 score (sexual function)	0(0, 0.1)	0(0, 1.0)	0.802
NMSS domain 9 score (miscellaneous)	1.0(0, 4.0)	1.0(0, 4.0)	0.570

The “*” mark denotes a significant *P* value (*P* < 0.05) after a Mann-Whitney test

Regarding the UPDRS score, the MDS-UPDRS part III, UPDRS tremor score, and UPDRS PIGD scores were numerically, but not significantly, higher in the MSD group compared to the non-MSD group after the Mann-Whitney *U* test (*P* > 0.05 for all).

In the univariate analysis of the NMSS scores, the Mann-Whitney *U* test revealed that both NMSS domain 4 (perceptual problem) and NMSS domain 5 (attention/memory) scores were significantly higher in the MSD group than in the non-MSD group (*P* = 0.033 and *P* < 0.001, respectively). However, there were no significant differences in the other NMSS domain scores between the two groups (*P* > 0.05 for all).

### Multivariate logistic regression

At first, 10 significant factors resulted from the t-tests, Mann-Whitney *U* tests and Chi-squared tests, including educational level, occupation, monthly income, speaking background, frontal lobe lesions, temporal lobe lesions, concurrent dysphagia, ADL score, NMSS domain 4 score (perceptual problems) and NMSS domain 5 score (attention/memory) were included for multivariate logistic regression analysis. To reduce the potential FWER attributable to t-tests and Mann-Whitney *U* tests, a Bonferroni correction of *P*-value (0.05/10 = 0.005) was reviewed to be a statistical significance. After the multivariate logistic regression analysis, 7 factors were excluded from the pool of independent risk factors. On the other hand, potential risk factors, including educational level, frontal lobe lesions, and NMSS domain 5 score (attention/memory), had a significant power to predict risk for MSD in PD (see Table 4).

**Table 4:** Potential independent risk factors for PD-associated MSD after the multivariate logisticregression analysis

Variables	OR(95% CI)	*P* value
Educational level	0.674(0.590, 0.808)	0.002
Frontal lobe lesions	5.145(2.018, 7.308)	0.004
NMSS domain 5 score (attention/memory)	10.458(6.164, 15.209)	0.001

The patients with a higher educational level [OR = 0.674, 95% CI (0.590, 0.808)] had a lower likelihood of PD-associated MSD. However, patients with a higher occurrence of frontal lobe lesions [OR = 5.145, 95% CI (2.018, 7.308)], or higher NMSS domain 5 scores (attention/memory) [OR = 10.458, 95% CI (6.164, 15.209)] had a higher likelihood of PD-associated MSD.

## Discussion

This study comprehensively explored the independent risk factors, including demographic and clinical characteristics and relevant PD-associated MSD evaluations for the first time. Univariate analysis demonstrated that the MSD phenotype was associated with a lower educational level and a greater amount of manual labor, and a monolingual speaking background. These findings were partially in accordance with those of similar studies that focused mainly on the demographic characteristics and relevant evaluations in other types of SD [22–25]. Moreover, multivariate logistic regression analysis revealed a lower educational level as an independent risk factor for PD-associated MSD. Several studies on the neural mechanisms underlying motor speech control [15, 16, 26, 27] have hypothesized that a higher educational level might play a negative role in the occurrence and aggravation of PD-associated MSD. Previous MRI examinations have shown that lesions in cognitive-relevant cortical areas, especially those closely associated with educational level within the prefrontal lobe, frequently occur in SD patients [28–30]. Given that educational level is usually positively associated with high-level cognitive function, this finding might partially explain why there were more PD patients with low educational levels in the MSD group in the present study [31–34].

Additionally, both univariate and multivariate logistic regression analyses revealed a higher occurrence of frontal lesions in the MSD group, indicating that frontal lesions are another important independent risk factor for PD-associated MSD. This is could also explain our speculation that PD patients in the MSD group were more prone to cognitive impairment since frontal lesions have been reported as a risk factor for the development of cognitive dysfunction [35–38]. Additionally, the higher occurrence of frontal lesions in the MSD group might also be attributed to a lack of sufficient plastic repair in some cortical areas following PD, especially in the frontal lobe, a well-known key area for central modulation during speech motor processing. On the other hand, secondary damage remote from the basal ganglia, an indispensible fiber pathway and subcortical interconnecting structure, could occur in the frontal lobe [39–41]. Therefore, future prospective clinical and neuroimaging studies are necessary to confirm these relationships and provide new insights into the neuroplastic repair in cortical areas.

Previous studies have systematically explored the relationship between MSD and cardinal motor symptoms. Moustafa et al. found that motor symptoms such as gait disturbance share similar clinical profiles and neural bases with MSD [42]. Majdinasab et al. found that tremor was the only aspect of motor symptoms that influenced MSD among PD patients [43]. However, in the present study, the UPDRS motor sum, tremor, and PIGD score were all ruled out following the univariate analysis. The difference between our study and that of Majdinasab et al. might be in the different disease stages. Patients in the previous study were predominantly in the early stage of PD, when motor symptoms are common, while our patients were in more progressive stages of PD. Additionally, both MSD and other motor symptoms are partly under the modulation of the extrapyramidal system, which causes similar neuropathological changes.

In the present study, univariate analysis showed that the NMM burden was slightly greater in the MSD group than in the non-MSD group, which was consistent with our speculation that MSD and NMM share similar neuropathological mechanisms. We found that the MSD group had a higher NMMSS domain 5 score (attention/memory) than the non-MSD group. Cognitive function impairment, including attention, memory, and executive functions, are common in PD patients; therefore, the cause and effect relationship between cognitive impairment and MSD requires in-depth examination. In terms of neural substrates, the frontal lobe is predominantly considered as the key modulation area responsible for the neural coding of high-level cognitive function and as a positive influencer of cognitive integration, which explains the attention and memory impairment in the MSD group [44–46]. In addition to the anatomical findings, we also speculate that MSD and cognitive impairment might share a similar pathophysiological process. Consistent with several previous findings, we found that the invalidation of anti-PD medication was more remarkable in PD patients who developed MSD and other NMMs than in tremor-dominant patients. These findings indicate that the root cause of MSD and other NMMs might involve non-dopaminergic neurons and non-dopaminergic transmitter disorders, such as long-term neurodegeneration induced by a gradual loss of cholinergic neurons [10, 12, 47]. Generally, we reasoned that the MSD group was likely to experience more cognitive dysfunction since speech motor modulation and cognitive functions are likely to share common neural anatomy and neurodegenerative processes. In the present study, attention/memory impairment was retained after removal of the potential risk factor (perceptual problems) from the multivariate logistic regression analysis, suggesting that there may be a strong correlation between high-level cognitive function (especially attention and memory) and MSD in PD patients. This finding demonstrated that a detailed scale for neuroimaging evaluation of attention and memory, as well as valid cognitive treatment for the improvement of attention and memory, might improve the prognosis of MSD in PD patients. Moreover, determining the cause and effect relationship between MSD and attention/memory impairment in PD could help determine the detailed neurophysiologic and neuropathological mechanisms underlying the abnormal speech motor modulation. In addition, although we evaluated attention and memory as two separate high-level cognitive functions, it was difficult to separate the two different cognitive subtypes in the MSD and non-MSD groups. This could be attributed to the fact that a majority of the patients manifested with mixed dysfunction of the two cognitive subtypes, or that speech modulation involves the two cognitive subtypes. Nonetheless, we have to acknowledge that we just assessed patients’ attention or memory capacity via a single subjective question in NMSS, respectively. Therefore, lack of some direct objective cognition tests might bring about some limitation to present study. In the future study, we plan to carry out some direct objective cognition measurements to further explore the potential relationship between cognition and MSD in PD patients.

Several different studies have reported that speech is a multisensory process in which auditory perception plays a key role [48–51]. Additionally, some studies have reported that MSD patients are more likely to suffer more from perceptual disorder; however, the elaborate relationship and mechanism involved in PD-associated perceptual problems are unknown [52–54]. In the present study, univariate analysis showed a greater burden of perceptual problems in the MSD group than in the non-MSD group, but multivariate logistic regression analysis excluded perceptual problems as an independent risk factor for PD-associated MSD. However, as in the previous study, we did not explicitly define perceptual problems based on specific sensory function disorders. Therefore, studies with larger sample sizes and more comprehensive methods for evaluation of specific sensory disorders are necessary to confirm the relationship between perceptual problems and PD-associated MSD and to determine if more severe perceptual problems are related to a higher likelihood of MSD in PD patients.

### Limitations

Tough we have tried our best to improve our present study, there are still some limitation as follows. Firstly, as mentioned in Discussion section, we just evaluated attention/memory capacity by a single item subjective question, respectively. This might make our study less objective, in that cognition tests were missing. Secondly, there was a little shortage in our neuroimaging method. We have successfully prevented missing MRI data, however, we just measured the occurrence of lesion within any a lobe rather than the number or the volume of lesions. This might make our present study ambiguous that we have not appropriately utilized our MRI data. It is helpful for our addition of limitation to readers that they will be more objective to interpret our present study.

## Conclusions

We demonstrated more severe attention/memory function impairment in the MSD group compared to the non-MSD group. Additionally, for the first time, cognitive impairment was shown to be strongly correlated with MSD in PD patients. The early application of cognitive treatment may help improve rehabilitation management and quality of life in patients with PD-associated MSD. Moreover, future studies could further assess cognitive impairment to determine the detailed mechanisms underlying abnormal speech motor modulation in PD patients.

## Data Availability

The datasets used and/or analysed during the current study are available from the corresponding author on reasonable request.

## Abbreviations

ADL: Activities of daily living
FDA: Frenchay dysarthria assessment
MRI: Magnetic resonance imaging
MSD: Motor speech disorder
NMM: Non-motor manifestations
PD: Parkinson’s disease
PIGD: Postural instability gait difficulty
SD: Speech disorders
UPDRS: Unified Parkinson’s Disease Rating Scale
